# Divergent psychological stress response patterns to the COVID-19 pandemic in psychiatric patients with vs. without PTSD: A real-world exploratory study

**DOI:** 10.1371/journal.pone.0318839

**Published:** 2025-04-02

**Authors:** Marit Treptow, Claudia Bartels, Mirjana Ruhleder, Alexander Kratzenberg, Thorgund Reh-Bergen, Mona Abdel-Hamid, Luisa Heß, Jörg Signerski-Krieger, Katrin Radenbach, Björn-Hendrik Schott, Jens Wiltfang, Claus Wolff-Menzler, Ulrike Schmidt, Michael Belz

**Affiliations:** 1 Department of Psychiatry and Psychotherapy, University Medical Center Goettingen, Göttingen, Germany; 2 Department of Psychiatry and Psychotherapy, University of Duisburg-Essen, LVR-Hospital Essen, Essen, Germany; 3 German Center for Neurodegenerative Diseases (DZNE), Goettingen, Germany; 4 Leibniz Institute for Neurobiology, Magdeburg, Germany; 5 Department of Medical Sciences, Neurosciences and Signaling Group, Institute of Biomedicine (iBiMED), University of Aveiro, Aveiro, Portugal; 6 Department of Psychiatry and Psychotherapy, RG Molecular and Clinical Psychotraumatology & Psychotrauma, Treatment Unit, Rheinische Friedrich-Wilhelms University of Bonn, Bonn, Germany; 7 Department of Psychiatry and Neuropsychology, School for Mental Health and Neuroscience, Maastricht University Medical Centre, Maastricht, The Netherlands; Fondazione Policlinico Universitario Agostino Gemelli IRCCS, Universita' Cattolica del Sacro Cuore, ITALY

## Abstract

The COVID-19 pandemic has been shown to increase psychological burden and requires efficient coping strategies to maintain mental health. In particular, it remains unclear which pandemic-related stress response pattern occurs in pre-existing posttraumatic stress disorder (PTSD) during the pandemic – at the same time these patients potentially exhibit dysfunctional coping of artificially generated psychosocial stressors. To analyze this so far widely unconsidered pandemic-related stress response in PTSD, this study longitudinally measured psychosocial burden and adjustment disorder (AD) symptom load in 14 patients with a primary or secondary diagnosis of PTSD vs. a cohort of 145 psychiatric patients without PTSD. The previously established Goettingen psychosocial Burden and Symptom Inventory (Goe-BSI) was used. Patients were interviewed at the end of the first (April/May 2020) and the second nationwide lockdown in Germany (November/December 2020). In our convenience sample, psychiatric disorders were diagnosed by patients’ treating clinicians prior to study inclusion. Psychosocial burden and AD symptom load were significantly higher in patients with PTSD than in patients without PTSD over the course of the pandemic (both *p* = .005). Moreover, explorative analysis of Goe-BSI-assessed general psychiatric symptoms did not reveal changes during the pandemic in patients with PTSD. In sum, we provide preliminary evidence that, in relation to psychiatric patients without PTSD, those with PTSD might experience a higher pandemic-related burden and might thus cope less efficiently with this enduring real-world stressor. This study is limited inter alia by the small sample size and by the underrepresentation of some psychiatric diagnoses.

## Introduction

In February 2023, the World Health Organization [1] announced 6.8 million deaths and a rate of 756 million infections caused by the SARS-CoV-2 virus. The pandemic can be considered a strong global stressor due to the global health threat and resulting lockdown restrictions. As such it requires significant adaptation and endangers mental health particularly in those having low resilience.

A pandemic-related increase in first-onset mental illnesses in the general population has been described by several studies in different populations [2–11], including health-care professionals [4,5,12–16] and patients with COVID-19 [17,18]. For the period from March until May 2020 which covered a first lockdown in numerous countries, the COVID-19 pandemic has led to worsening of mental disorders as it is the case for eating disorders [19], bipolar disorder [20] as well as obsessive-compulsive disorder [21]. Moreover, independent from the presence of any psychiatric disorder, the pandemic-related stress response pattern included an increased level of depressive and anxiety symptoms during this early period of the pandemic [22]. Beyond that, the temporal dynamic of the pandemic – e.g., termination of lockdowns, variations of social restrictions – should be considered from a longitudinal perspective. In our previous study we could identify a specific stress response pattern: there, psychosocial burden for different pre-existing mental disorders did not exclusively worsen during the pandemic, but varied over time. We found an increase in psychosocial burden from pre-pandemic until Mid-March 2020, a steady decrease from Mid-March until April/May 2020 and a continued decrease until November/December 2020 [23]. Furthermore, patients with an ICD-10 F4-diagnosis reached the highest scores for all time-points over time compared to patients with other mental disorders.

An exposure-based intervention is the most effective therapy for PTSD [24], which usually could not be continued in presence during the first months of the pandemic due to lockdown restrictions. Many of those patients did not receive any treatment, or could merely be treated via telemedicine (telephone or video contacts). In general, people have experienced a lack of social support during the pandemic with increased feelings of loneliness [25,26]. This social support is a specifically important factor for patients with PTSD to cope with stress-related events [27]. In particular, social support enables for social discrimination learning, proactive coping, healthier behaviors, more positive mood and the release of oxytocin in patients with PTSD [28]. Oxytocin has been shown to increase feelings of security and to reduce the stress response in such patients [29] whereas social isolation, on the other hand, is associated with a negative neurobiological impact [30]. Additionally, symptoms of depression, anxiety, and acute stress which widely increased during the early phase of the pandemic (see above) are also posttraumatic factors with a potential to facilitate the development and/or increase the severity of PTSD [31]. In sum, patients with PTSD seemed specifically vulnerable during the nationwide lockdowns.

Of note we did not find any study specifically analyzing the pandemic-related stress response pattern caused by the impact of the COVID-19 pandemic on distress perception, psychosocial burden and psychiatric symptom load in patients suffering from posttraumatic stress disorder (PTSD). However, some studies could show that patients with pre-existing anxiety disorders (such as PTSD) reached a significantly higher rate of distress caused by self-isolation in comparison to patients with mood disorder and healthy controls [32]. In addition, a low level of social support combined with a high rate of pandemic-related stressors could increase the symptom severity of PTSD [33].

Beyond that, the prevalence of PTSD during or in response to the COVID-19 pandemic has been assessed in various populations. For instance, a meta-analysis reported an increased prevalence of PTSD in the general population (about 24%) after the beginning of the COVID-19 pandemic [3]. Furthermore, a web-based survey revealed a COVID-19-related PTSD prevalence of 29.5% in the Italian population and thus classified the pandemic as a traumatic stressor [34]. Accordingly, a recent study detected that about 75% of COVID-19 survivors in Manipur suffered from PTSD [35].

PTSD is a severe psychiatric syndrome that comprises aversive re-experiencing of trauma-related details, associated avoidance behavior, emotional numbing and nervous hyperarousal [36]. Several studies showed that psychological stress coping is impaired in patients with PTSD compared to healthy controls (e.g., [37,38]), possibly due to alterations in the regulation of the two major stress axes, the hypothalamic-pituitary-adrenal (HPA) axis (e.g., [38]) and the sympathetic nervous system [39]. Of note, PTSD and major depression were found to be predicted by different psychological stress coping styles [40] suggesting that pathological alterations in stress coping might differ among psychiatric disorders.

However, we found no study analyzing whether impaired stress coping in PTSD also occurs in response to enduring real-world stressors such as the COVID-19 pandemic and results in a higher symptom load. This motivated us to compare psychosocial burden and adjustment disorder (AD) symptom load in psychiatric patients with vs. without PTSD during the course of the pandemic using our previously established Goe-BSI inventory and our previously published cohort [23,41]. In this convenience sample, we previously found a specific stress response pattern: during the course of the pandemic, the initial increase in psychosocial burden was relieved while AD symptom load remained unchanged between the first (April, 24^th^ to May, 11^th^ 2020) and the second lockdown (November 27^th^ to December, 22^th^ 2020) in Germany [23]. So far, we concentrated on analyzing the total cohort of patients suffering from various mental disorders and did not focus on specific diagnostic subgroups such as patients with PTSD.

The aim of this study was to focus on patients with pre-existing PTSD and to compare them to a cohort of psychiatric patients with other pre-existing mental disorders, in order to analyze possible differences in the pandemic-related stress response pattern between both groups. To achieve this, psychosocial burden and symptoms of AD were measured at two different time-points during nationwide lockdown periods.

## Materials and methods

**Fig 1:**
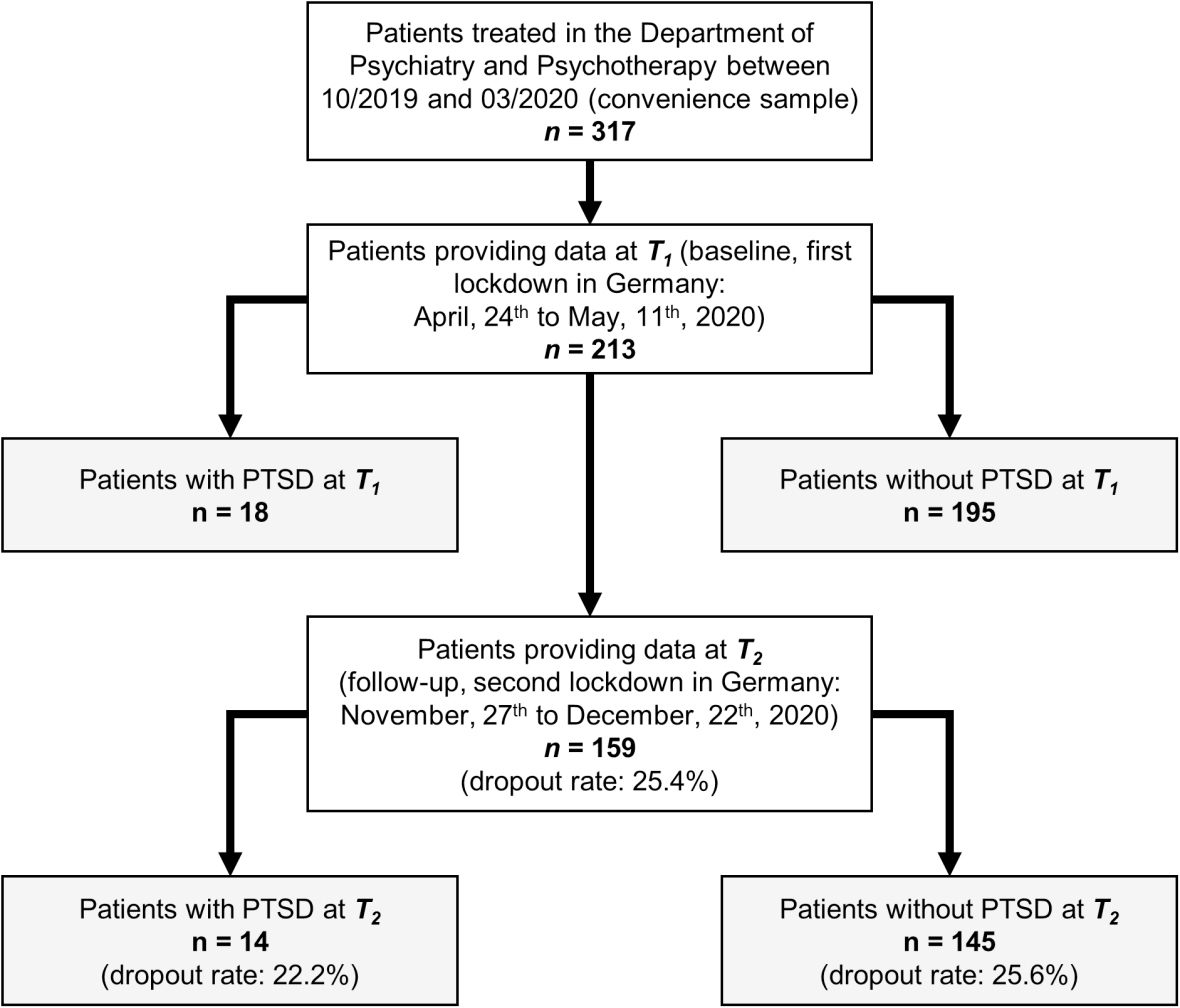
Selection of study patients.

**Fig 2:**
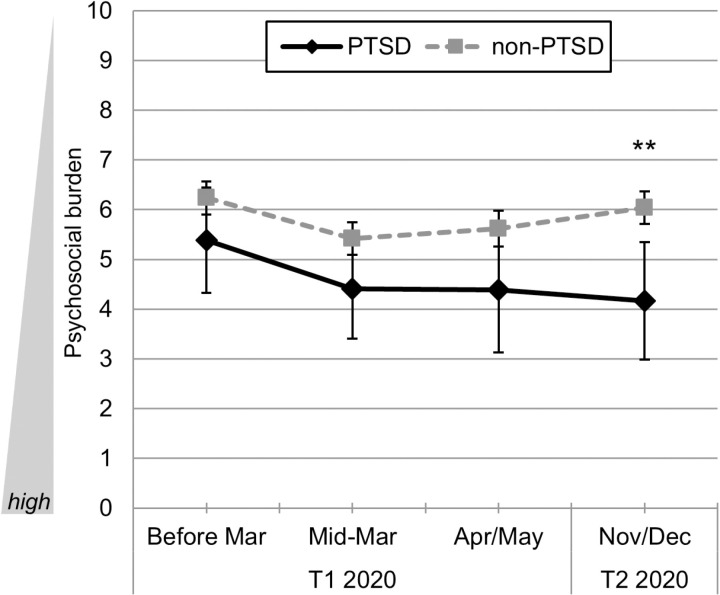
Course of psychosocial burden during different phases of the Covid-19 pandemic in psychiatric patients with PTSD (PTSD sample, ICD-10: F43.1, *n* = 14) vs. psychiatric patients without PTSD (non-PTSD sample, diagnosis other than ICD-10: F43.1, *n* = 142; *n* = 3 were excluded due to missing values). Mean values with 95%-CIs and Bonferroni-corrected pairwise comparisons. Psychosocial burden was assessed with the Goe-BSI and is presented as mean of ratings on the 11-point numeric scales for psychosocial stress, psychiatric symptomatology, and quality of life. Time-points of analyses: *T*_*1*_: *before* the pandemic (before March of 2020); at the *beginning* of the pandemic (mid-March 2020); first lockdown (April, 24^th^ to May, 11^th^ 2020); *T*_*2*_: second lockdown (November 27^th^ to December, 22^th^ 2020). Symbols: ****p**** <  0.05, ** ****p**** <  0.01, *** ****p**** <  0.001.

**Fig 3:**
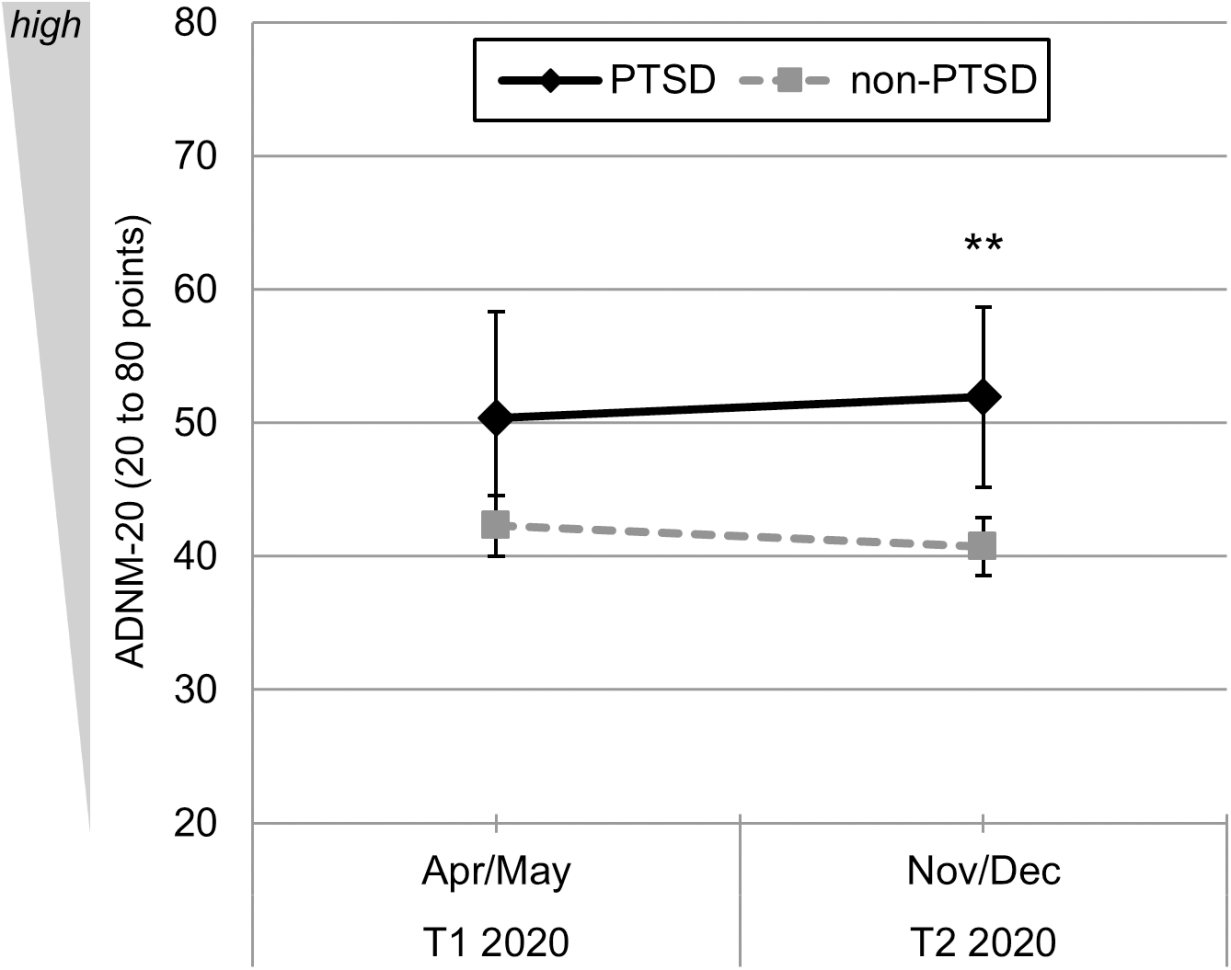
Course of symptom levels of AD during different phases of the Covid-19 pandemic measured with the ADNM-20 in psychiatric patients with PTSD (PTSD sample, ICD-10: F43.1, *n* = 14) vs. psychiatric patients without PTSD (non-PTSD sample, diagnosis other than ICD-10: F43.1, *n* = 138; *n* = 7 were excluded due to missing values). Mean values with 95%-CIs and Bonferroni-corrected pairwise comparisons. Symptom level of AD was assessed with the ADNM-20 sum score (range: 20 to 80 points). Time-points of analyses: *T*_*1*_: first lockdown (April, 24^th^ to May, 11^th^ 2020); *T*_*2*_: second lockdown (November 27^th^ to December, 22^th^ 2020). Symbols: ****p**** <  0.05, ** ****p**** <  0.01, *** ****p**** <  0.001.

Please note that further details are given in the legends of Tables 1–3, Figs 1–3.

**Table 1:** Comparison of general psychiatric symptoms and resilience during the first (*T*_*1*_: Q2/2020) vs. the second lockdown (*T*_*2*_: Q4/2020) in patients with PTSD.

*Goe-BSI items*	Apr/May 2020 (*T*_*1*_) *M* ± SD	Nov/Dec 2020 (*T*_*2*_) *M* ± SD	*p* ^1^
**(A) General psychiatric symptoms**			
1. “I have become more vigilant than before the corona-crisis.”	5.36 ± 3.67	6.50 ± 2.25	.283
2. “Since the beginning of the crisis, I have spent more time on the internet or with media than before (except for home office).”	4.79 ± 3.60	3.07 ± 3.32	*.080*
3. “I don’t enjoy things the way I used to since the beginning of the crisis.”	4.21 ± 2.19	4.50 ± 3.37	.738
4. “Feelings of anxiety have increased since the crisis began.”	4.00 ± 2.54	3.62 ± 3.25	.589
5. “Due to the crisis, I have less drive to undertake and tackle things.”	3.71 ± 2.30	4.43 ± 3.23	.260
6. “Since the beginning of the crisis, I have increasingly withdrawn emotionally from others. (not meant: social distancing).”	3.57 ± 2.93	3.64 ± 3.80	.938
7. “Since the beginning of the corona-crisis, I have been less physically active.”	3.14 ± 2.91	4.29 ± 3.47	.306
8. “Since the beginning of the corona-crisis, I have had more physical symptoms than before.”	2.93 ± 3.32	1.77 ± 2.80	.265
9. “I have been paying more attention to possible symptoms of illness in others since the crisis began.”	2.79 ± 2.64	4.36 ± 2.90	*.092*
10. “I feel more anger or I am more aggressive since the crisis began.”	2.71 ± 2.79	3.79 ± 3.58	.193
11. “My cognitive functions (orientation, comprehension, concentration, memory) have declined during the corona-crisis.”	2.71 ± 3.41	3.14 ± 3.46	.701
12. “I have been paying more attention to possible symptoms of illness in myself since the crisis began.”	2.57 ± 3.03	3.86 ± 3.18	*.095*
13. “Since the beginning of the crisis, I eat more or less than before.”	1.86 ± 2.80	1.36 ± 2.71	.511
14. “Compared to the time before the crisis, I have more conflicts with other people.”	1.64 ± 1.95	2.57 ± 3.20	.314
15. “Since the beginning of the crisis, I have felt watched and persecuted more often.”	1.57 ± 2.31	1.93 ± 2.59	.671
16. “Since the beginning of the crisis, I have been engaging in self-injurious behavior more frequently.”	1.14 ± 2.93	0.86 ± 2.18	.486
17. “Since the beginning of the crisis, I have more often special perceptions that others do not have (e.g., hearing voices, seeing people or things).”	1.00 ± 2.42	0.00 ± 0.00	.146
18. “Since the beginning of the crisis, I have felt more strongly that others are conspiring against me.”	1.00 ± 1.30	0.93 ± 2.20	.916
19. “Since the beginning of the crisis, I have been taking more pills.”	0.93 ± 1.90	0.14 ± 0.54	.174
20. “Compared to the time before the crisis, physical or psychological violence in my partnership or family has increased.”	0.64 ± 1.65	1.29 ± 3.12	.168
21. “Since the beginning of the crisis, I have had greater craving for addictive substances (alcohol, illicit drugs) than before.”	0.36 ± 1.34	0.64 ± 1.15	.470
22. “Since the beginning of the crisis, I consume more alcohol or illicit drugs.”	0.14 ± 0.54	0.07 ± 0.27	.336
**(B) Resilience**			
1. “For me, some things have changed in a positive way during the pandemic.”	4.00 ± 3.42	4.21 ± 3.89	.847
2. “The pandemic also holds opportunities for me.”	2.50 ± 3.23	2.86 ± 3.53	.707

English translation of the Goe-BSI (Goettingen psychosocial Burden and Symptom Inventory) items regarding **(A)** general psychiatric symptoms and **(B)** resilience with means (*M*), and standard deviations ( ± SD) for *T*_*1*_: (first lockdown in Q2/2020) and *T*_*2*_ (second lockdown in Q4/2020). All items were rated on a scale from 0 to 10 (0 =  “does not apply at all” to 10 =  “fully applies”). The mean values gained at *T*_*1*_ were sorted by size in descending order.

^1^Uncorrected *p*-values (t-tests for repeated measures; *N* =  13-14; *df* =  12-1

**Table 2:** Correlations between diagnostic groups, sociodemographic variables and general psychiatric symptoms and resilience (secondary endpoints).

*Variable*	1	2	3	4	5	6	7	8	9	10	11
**Diagnostic and sociodemographic variables**											
1. PTSD (F43.1) vs. non-PTSD (other F-diagnosis)	–										
2. Age (in years)	−0.025	–									
3. Sum of F-Diagnoses	0.284^**^	−0.031	–								
4. Living space (in m²)	−0.196^*^	0.101	−0.133	–							
5. Covid-19 risk group (yes:no; %)	−0.101	−0.492^**^	−0.102	−0.010	–						
**Most pronounced psychiatric symptoms at *T***_***2***_											
6^1^. Vigilance	0.103	0.037	0.186^*^	0.058	−0.173^*^	–					
7^1^. Loss of joy	0.161^*^	0.153	0.164^*^	0.018	−0.119	0.424^**^	–				
8^1^. Poor drive	0.083	0.121	0.207^**^	−0.092	−0.142	0.477^**^	0.707^**^	–			
9^1^. Observing disease symptoms of others	−0.003	−0.049	0.148	0.219^**^	0.002	0.478^**^	0.232^**^	0.301^**^	–		
10^1^. Being less physically active	0.032	0.041	0.113	−0.054	−0.096	0.366^**^	0.436^**^	0.514^**^	0.243^**^	–	
**Resilience at *T***_***2***_											
11^2^. Positive changes	0.028	−0.271^**^	−0.065	−0.063	0.128	−0.069	−0.223^**^	−0.205^*^	0.118	−0.387^**^	–
12^2^. Opportunities	0.014	−0.199^*^	−0.036	0.059	0.116	−0.081	−0.258^**^	−0.230^**^	0.086	−0.367^**^	0.631^**^

Correlations:

*  *p* <  0.05.

** *p* <  0.01. Captions: *F43.1* (*PTSD* =  1, *other F-diagnosis* =  0); self-reported *risk group* status for a severe course of Covid-19 (yes =  1, no =  2); variables measured at *T*_*2*_ (follow-up: second lockdown Q4/2020):

^1^top-five psychiatric symptoms (most pronounced in patients with PTSD): items rated from 0 to 10;

^2^resilience: items rated from 0 to 10. (*N* =  146; *df* =  144 to *N* =  159; *df* =  157). Note that numbers in columns refer to numbers in rows.

**Table 3:** Comparison of sociodemographic variables in the PTSD sample vs. the non-PTSD sample at baseline (*T*_*1*_: Q2/2020).

	Total sample (*N* = 159)	PTSD sample (*n* = 14)	Non-PTSD sample (*n* = 145)	Valid *N*	*p* ^1^
**Sociodemographic variables**					
1. Age	*M* = 41.13 ± 15.95	*M* = 39.86 ± 13.42	*M* = 41.25 ± 16.21	159	.756^1a^
2. Gender binary (male:female; %)	72:64 (52.9%, 47.1%)	4:9 (30.8%, 69.2%)	68:55 (55.3%, 44.7%)	136	*.092* ^1c^
3. Sum of F-diagnoses	*M* = 1.84 ± 1.04	*M* = 2.79 ± 1.42	*M* = 1.74 ± 0.96	159	*.001* ^***,1a^
4. Living space (in m^2^)	*M* = 92.47 ± 53.53	*M* = 58.69 ± 26.83	*M* = 95.70 ± 54.38	149	*.017* ^*,1a^
5. Persons in household	*M* = 2.28 ± 1.97	*M* = 1.64 ± 1.01	*M* = 2.34 ± 2.03	159	.204^1a^
6. Covid-19 risk group (yes:no; %)	55:104 (34.6%, 65.4%)	7:7 (50.0%, 50.0%)	48:97 (33.1%, 66.9%)	159	.204
**Changes from first to second lockdown**					
7. Tested for Covid-19 (yes:no; %)	51:107 (32.3%, 67.7%)	6:8 (42.9%, 57.1%)	45:99 (31.3%, 68.8%)	158	.375^1b^
8. Change in marital status (yes:no; %)	8:148 (5.1%, 94.9%)	1:13 (7.1%, 92.9%)	7:135 (4.9%, 95.1%)	156	.537^1c^
9. Change in living situation (yes:no; %)	20:136 (12.8%, 87.2%)	2:11 (15.4%, 84.6%)	18:125 (12.6%, 87.4%)	156	.674^1c^
10. Change in work situation (yes:no; %)	35:124 (22.0%, 78.0%)	3:11 (21.4%, 78.6%)	32:113 (22.1%, 77.9%)	159	.999^1c^
11. Kind of change: work situation (pos.:neg.; %)	16:17 (48.5%, 51.5%)	1:2 (33.3%, 66.7%)	15:15 (50.0%, 50.0%)	33	.999^1c^
12. Change in financial situation (yes:no; %)	30:128 (19.0%, 81.0%)	4:10 (28.6%, 71.4%)	26:118 (18.1%, 81.9%)	158	.307^1c^
13. Kind of change: financial situation (pos.:neg.; %)	13:17 (43.3%, 56.7%)	2:2 (50.0%, 50.0%)	11:15 (42.3%, 57.7%)	30	.999^1c^
14. Change in childcare (yes:no; %)	15:142 (9.6%, 90.4%)	3:11 (21.4%, 78.6%)	12:131 (8.4%, 91.6%)	157	.135^1c^

Data presented as means (*M*), standard deviations ( ± SD), and frequencies. Captions: *Gender* (binary: male =  1, female =  2); *risk group* for a severe course of Covid-19 (yes =  1, no =  2);

^1^uncorrected *p*-values for metric variables (^1a^*t*-tests), and binary variables (^1b^2×2 ꭓ^2^-tests, or ^1c^Fisher’s exact test for cells <  *n* =  5). Valid percentages are presented (please see *Valid N* for the available number of cases for each variable).

### Study sample

For the total sample recruited at baseline (*T*_*1*_: first lockdown, April, 24^th^ to May, 11^th^ 2020 [41], we applied the following inclusion criteria: (1) patients who were ≥  18 years old, (2) patients who were treated in the Department of Psychiatry and Psychotherapy of the University Medical Center (UMC) Göttingen, Germany between 10/2019 to 03/2020, (3) patients who received no hospitalization at the time-point of inclusion, (4) patients with presence of at least one ICD-10 mental/psychiatric diagnosis (F00-F99) at the time-point of inclusion, (5) patients with capacity for providing informed consent and study data. As we reported previously, of 213 patients at baseline (*T*_*1*_), 159 patients took part in follow-up-measurements (*T*_*2*_; second lockdown, November 27^th^ to December, 22^th^ 2020 [23]; dropout rate of 25.4%). At *T*_*1*_, due to pandemic-related contact restrictions, written informed consent could not be obtained in person but only by mail. Only those patients that had already given consent at *T*_*1*_ were contacted again at *T*_*2.*_ In general, informed consent was obtained for study participation and anonymized publication of individual data. In order to comply with the applicable data protection regulations, the data were analyzed in pseudonymized form. First, each study participant was assigned a unique identification number. Subsequently, these identification numbers were converted into consecutive numbers by a person independent of the project. Personal data for re-contacting were stored on a separate list. This list was stored in a location physically accessible only to the study director and his deputy. Beyond this, the authors did not have access to any information that could identify individual participants.

### Study design

After ethical approval by the Ethics Committee of the UMC Goettingen (#36/4/20), all interviews were performed by phone in German language and were conducted at two time-points during the COVID-19 pandemic in Lower Saxony, Germany, i.e., at *T*_*1*_ (April, 24^th^ to May, 11^th^ 2020) and *T*_*2*_ (November 27^th^ to December, 22^th^ 2020; see Fig 1 for details). Interviews were performed by 28 qualified and specialized clinicians (psychologists/psychotherapists and psychiatrists) who all received a rater training before the start of assessments. ICD-10 diagnoses of included patients were established during clinical routine by their treating clinicians (residents, board-certified psychiatrists, psychologists, or licensed psychotherapists). For the analyses presented here, we extracted a PTSD subsample from our cohort: in case PTSD (ICD-10: F43.1) was diagnosed as primary or secondary diagnosis, patients were assigned to the PTSD sample while all remaining cases constitute the non-PTSD sample (please see results section for details). All patients with suspected PTSD were diagnosed with trauma-specific instruments (interviews and questionnaires) using the Essen Trauma-Inventory (ETI) [42] and the Impact of Event Scale - Revised (IES-R) [43].

### Study measures

The Goettingen psychosocial Burden and Symptom Inventory (Goe-BSI), a standardized and structured telephone interview, was applied at *T*_*1*_ and *T*_*2*_ – please see our previous publications for detailed information about scale development, quality criteria and further details [23,41]. Besides demographic data, the Goe-BSI allows assessment of the course of general psychiatric symptoms during the pandemic along with resilience. It is, so far, exclusively available in German language. We defined two primary outcome measures to operationalize stress coping in PTSD, namely (1) psychosocial burden and (2) the presence of AD symptoms in response to the pandemic (AD New Module – 20 Item version [ADNM-20] [44]).

Briefly, the assessment of psychosocial burden consisted of three items rated on 11-point numeric scales which were formulated as questions: psychosocial stress, psychiatric symptoms, and quality of life with lower scores indicating higher psychosocial burden (0: It could not be worse; 10: It could not be better). At *T*_*1*_, not only the current state was assessed but also, retrospectively, the states before and at the beginning of the pandemic when maximum pandemic-related restrictions had become active in Germany (first lockdown, mid-March, 2020). This resulted in four ratings per patient (three at *T*_*1*_, one at *T*_*2*_) and allowed to generate a trajectory of psychosocial burden from baseline to follow-up. The ADNM-20 was used at *T*_*1*_ and at *T*_*2*_ (current states only, no retrospective assessments) to measure psychological reactions to stressful life events (here: the pandemic). The ADNM-20 consists of 20 four-point items; 48 out of 80 points are defined as cut-off reflecting a high risk for the presence of an AD [45]. Assessment of psychiatric symptoms (20 items/statements) and resilience (two items/statements) was performed at both, *T*_*1*_ and *T*_*2*_. The spectrum of possible answers ranged from 0 (*strongly disagree*) to 10 (*strongly agree*) – please see Table 1 for all translated items).

### Statistical analyses

IBM SPSS Statistics, version 29 was employed for data analysis. Frequencies, means (*M*), standard deviations (±/ SD), and correlations (depending on scale level: Pearson correlations (metric), mean square contingency/phi coefficients (binary) – see Table 2) were computed for descriptive representation of metric variables.

Two general linear models for repeated measurements (GLM) were calculated for the two primary outcomes of this study, i.e., psychosocial burden and symptoms of AD (i.e., ADNM-20 sum score). For psychosocial burden, multiple measurements were added as four-staged within-subjects factor: each participant contributed three measurements at baseline (*T*_*1*_: *before* the pandemic, *at the beginning* of the pandemic, at *current state* during first lockdown), and one measurement at follow-up (*T*_*2*_: *current state* during second lockdown). A two-staged between-subjects factor was also added (PTSD sample vs. non-PTSD sample). For symptoms of AD (ADNM-20), two repeated measurements were included (*T*_*1*_ and *T*_*2*_: current state during first/second lockdown). Again, allocation to the PTSD sample was added as two-staged between-subjects factor. Besides main effects, the interaction effect (PTSD status ×  repeated measurement) was tested in each GLM to map possible different trajectories between patients with vs. without PTSD on both primary outcomes. Pairwise comparisons (*t*-tests) were then used to validate possible interaction effects. Missing data can be derived from degrees of freedom for each model. For multiple comparisons, *p*-values were corrected within each GLM, using the Bonferroni method (initial significance: *p* <  0.05, two-tailed).

For explorative analysis of psychiatric symptoms and resilience from *T*_*1*_ to *T*_*2*_, multiple t-tests for dependent measures were used without Bonferroni-correction (please see results section for details, Table 1).

## Results

### Demographical and clinical characteristics of the study sample

In total, 159 patients treated in the Department of Psychiatry and Psychotherapy of the UMC Goettingen completed the Goe-BSI by interview twice, i.e., both during the first (baseline: April, 24^th^ to May, 11^th^ 2020, *T*_*1*_) and the second (follow-up: November 27^th^ to December, 22^th^ 2020, *T*_*2*_) national lockdown phase. Out of these 159, 14 patients were assigned to the PTSD sample, either due to their main diagnosis (*n* =  7, 50.0%) or secondary diagnosis (*n* =  7, 50.0%) which had been determined by the patients’ treating clinicians (see material and methods). The remaining patients, i.e., the non-PTSD sample, consisted of *n* =  145 patients. The five most frequent main psychiatric ICD-10 diagnoses of this sample were (1) affective disorders (F3, *n* =  61, 42.1%), (2) disorders of adult personality and behavior (F6, *n* =  25, 17.2%), (3) schizophrenia, schizotypal and delusional disorders (F2, *n* =  21, 14.5%), (4) neurotic, stress-related and somatoform disorders (F4, *n* =  17, 11.7%), and (5) disorders of psychological development (F8, *n* =  14, 9.7%). The enrichment of the latter can be explained by the fact that our Autism Spectrum Outpatient Unit participated in recruitment of study subjects.

Table 3 shows sociodemographic variables of the PTSD in comparison to the non-PTSD sample. Two variables differed significantly between the PTSD and the non-PTSD sample, namely living space and sum of F-diagnoses: in comparison to psychiatric patients without PTSD, those suffering from this stress-related disorder had smaller living spaces (*M* =  58.69 m² ±  26.83 vs. *M* =  95.70 m² ±  54.38, *p* = .017) and a higher number of F-diagnoses (*M* =  2.79 ±  1.42 vs. non-PTSD sample: *M* =  1.74 ±  0.96, *p* < .001). The latter suggests a comparably higher level of comorbidity in PTSD patients. The number of persons of female gender were elevated in the PTSD sample with a trend for statistical significance (*p* = .092; PTSD: *n* =  9 female (64.3%), *n* =  4 male (28.6%), *n* =  1 patient of non-binary gender (7.1%) vs. non-PTSD: *n* =  55 female (44.7%), *n = * 68 male (46.9%), *n* =  22 of non-binary gender (15.2%)). In the PTSD vs. the non-PTSD sample there were no differences in the mean age (PTSD: *M* =  39.86 years, SD =  13.42, range: 23 to 70 years; non-PTSD *M* =  41.25 years, SD =  16.21, range: 18 to 82 years). Half of the patients with PTSD reported that they belonged to a risk group for a severe course of a SARS-CoV-2 infection (*n* =  7), whereas this was the case in only 33.1% (*n* =  48) of those without PTSD (*ns*). Until the time-point of the second lockdown, *n* =  6 of the PTSD sample (42.9%) vs. *n* =  45 of the non-PTSD sample (31.3%) had been tested for COVID-19 at least once (no positive results, no quarantine; *ns*). The majority of patients with and without PTSD reported that during the pandemic no changes had occurred in the following areas: family status, living condition, work situation, financial situation and childcare – there were no significant between-group differences in these parameters.

### Course of psychosocial burden

The GLM revealed a significant variation in psychosocial burden over time (total score of the Goe-BSI) for the total sample (*F*(3, 462) =  3.67, *p* = .012, partial η^2^ =  0.023): As we found previously [23], psychosocial burden significantly increased from the time-point before the pandemic/lockdown to its beginning, but then decreased during the first and second lockdown in the total cohort. Comparing the PTSD to the non-PTSD subsample, a significant between subjects-effect with a medium effect size was found (*F*(1, 154) =  8.02, *p* = .005, partial η^2^ =  0.05): Patients with PTSD showed comparably higher levels of psychosocial burden for the global set of measurement points (Fig 2). Although we did not find a significant interaction effect (*F*(3, 462) =  1.09, *ns*), descriptive analysis suggested that patients with PTSD showed an increase of psychosocial burden from the time-point *before* the pandemic (*M* =  5.38, SD =  2.02) to its *beginning* (*M* =  4.40, SD =  1.92), to the first lockdown (*T*_*1*_: *M* =  4.38, SD =  2.39) and to the second lockdown (Fig 2, *T*_*2*_: *M* =  4.17, SD =  2.25). In comparison, descriptive analysis of patients without PTSD also showed an increase of psychosocial burden from the time-point *before* the pandemic (*M* =  6.24, SD =  2.01) to its *beginning* (*M* =  5.42, SD =  1.98), followed by a decrease of psychosocial burden at the first lockdown (*T*_*1*_: *M* =  5.62, SD =  2.19), followed by an additional decrease at the second lockdown (Fig 2, *T*_*2*_: *M* =  6.04, SD =  1.97). While pairwise comparisons were non-significant for the first three time-points after Bonferroni-correction (*t* between 1.54 and 1.98, *ns*), we found a significant difference with a large effect size during the second lockdown (Fig 2, *current state T*_*2*_: *t*(155) =  3.34, *p* = .004, *d*_*emp*_ =  0.88) indicating a comparably higher psychosocial burden in the patient group with PTSD at the last time-point. Please see Table 4 for all mean values and differences between both subsamples.

**Table 4:** Stress response patterns between the PTSD sample and the non-PTSD sample (*T*_*1*_: Q2/2020, *T*_*2*_: Q4/2020).

	*T*_*1*_: before Mar	*T*_*1*_: mid-Mar	*T*_*1*_: Apr/May	*T*_*2*_: Nov/Dec
**Psychosocial burden**				
1. PTSD sample	*M* = 5.38 ± 2.02	*M* = 4.40 ± 1.92	*M* = 4.38 ± 2.39	*M* = 4.17 ± 2.25
2. Non-PTSD sample	*M* = 6.24 ± 2.01	*M* = 5.42 ± 1.98	*M* = 5.62 ± 2.19	*M* = 6.04 ± 1.97
3. Difference	*M*_*Diff*_ = −0.86	*M*_*Diff*_ = −1.02	*M*_*Diff*_ = −1.24	*M*_*Diff*_ = −1.87
**ADNM-20**				
1. PTSD sample			*M* = 50.36 ± 15.12	*M* = 51.93 ± 12.86
2. Non-PTSD sample			*M* = 42.27 ± 13.63	*M* = 40.70 ± 13.12
3. Difference			*M*_*Diff*_ = 8.09	*M*_*Diff*_ = 11.23

Data presented as means (*M*), standard deviations ( ± SD), and mean differences between PTSD-sample and non-PTSD sample (*M*_*Diff*_). Captions: Psychosocial burden is presented as mean of ratings on the 11-point numeric scales for psychosocial stress, psychiatric symptomatology, and quality of life (low values indicate high psychosocial burden). Symptom level of adjustment disorder was assessed with the ADNM-20 sum score (range: 20 to 80 points; high values indicate high symptom load). Time-points of measurements: *T*_*1*_: before the pandemic (before March of 2020); at the beginning of the pandemic (mid-March 2020); first lockdown (April, 24th to May, 11th 2020); *T*_*2*_: second lockdown (November 27th to December, 22th 2020).

### Symptoms of AD

As we reported previously [23], the GLM did not reveal a significant change in the ADNM-20 sum score from first to second lockdown in the total sample (*F*(1, 150) = .00, *p* = .998, *ns*). Focusing again on differences between the PTSD and non-PTSD subsample, we found a significant between subjects-effect with a medium effect size (*F*(1, 150) =  8.03, *p* = .005, partial η^2^ =  0.05): Symptoms of AD were generally more pronounced in patients with PTSD than in patients without PTSD for the global set of time-points (Fig 3). Again, we did not find a significant interaction effect (*F*(1, 150) =  0.95, *ns*). However, descriptive analysis suggested that patients with PTSD had an increase in AD symptoms from the first (*T*_*1*_: *M* =  50.36, SD =  15.19) to the second lockdown (Fig 3, *T*_*2*_: *M* =  51.93, SD =  12.86) whereas patients without PTSD had a decrease in AD symptoms (Fig 3, *T*_*1*_: *M* =  42.27, SD =  13.63; *T*_*2*_: *M* =  40.70, SD =  13.12). Bonferroni-corrected pairwise comparisons showed no significant difference during the first lockdown (*T*_*1*_: *t*(150) =  2.09, *p* = .076) but revealed a significant difference during the second lockdown, with a large effect size (*T*_*2*_: *t*(150) =  3.06, *p* = .005, *d*_*emp*_ =  0.86). Please see Table 4 for all mean values and differences between both subsamples.

### Exploratory analysis: Course of general psychiatric symptoms and resilience

Please see Table 2 for correlations between the diagnostic status of PTSD (yes/no) and (1) baseline sociodemographic variables in the total sample, (2) the five mostly pronounced psychiatric symptoms at second lockdown (*T*_*2*_), and (3) both resilience items. Three out of eleven correlations reached significance: in comparison with other diagnoses, PTSD status correlated directly with sum of F-diagnoses (Table 2, *r* =  -0.284, *p* < .001), inversely with living space (*r* =  -0.196, *p* = .017), and directly with loss of joy (*r* =  0.161, *p* = .043). However, following the definition by Cohen (1988), these *r*-values are to be interpreted between “small” (*r* =  0.10) and “medium” (*r* =  0.30) [46].

The development of a total of 22 pre-defined psychiatric symptoms was analyzed in the PTSD sample between the first (*T*_*1*_) vs. the second lockdown (*T*_*2*_) – please see Table 1 for all pairwise comparisons and items. In sum, analyses did not reveal any significant difference for these items; however, a trend (*p* < .100) could be found for three variables: (1) the time spent with internet/media decreased from first to second lockdown (*p* = .080), (2/3) both, the attention to symptoms of illness in others and the patients themselves, increased from first to second lockdown (*p* = .092 and .095). We found no effect of the ongoing pandemic on subjectively perceived resilience as both items did not differ between *T*_*1*_ and *T*_*2*_ in patients with PTSD (Table 1, *p* = .847 and.707).

## Discussion

To the best of our knowledge, this is the first study directly comparing the influence of the COVID-19 pandemic on psychosocial burden and AD symptom load as measures of stress coping in psychiatric patients with vs. without PTSD. This finding is of clinical relevance since, although both PTSD and AD belong to the group of stress-related disorders in the ICD-10, they have clear diagnostic boundaries and require different types of treatment [47]. In summary, we found that, in relation to psychiatric patients without PTSD, the pandemic-related stress response pattern of patients with PTSD showed both a significantly higher psychosocial burden and AD severity during the COVID-19 pandemic.

Interestingly a deviation in pandemic-related stress response pattern was found between the subgroups. Psychosocial burden and AD severity differed significantly between both samples during the latest time-point during the pandemic examined here (second lockdown): While both outcomes are suggestive of an improvement for patients without PTSD, they seem to relatively aggravate in those with this type of stress-related disorder. This might suggest that, among adult psychiatric populations, patients with PTSD have a particularly high stress vulnerability and/or a particularly impaired stress coping which might, in turn, contribute to a possibly reduced resilience as described in previous literature (e.g., [26]). Specifically, the latter hypothesis is in line with previous findings showing that coping of various stressors is reduced in individuals suffering from PTSD (e.g., [38]) and that stress coping abilities differ between various psychiatric disorders (e.g., [37,40]). Accordingly, stress-coping styles have been found altered in PTSD [48,49]. Interestingly, several studies suggest that the time-point of traumatization, in particular traumata survived in childhood vs. those experienced in adulthood, highly likely modulates the psychological and/or molecular stress response in PTSD [38,50]. Thus, for future experiments, it would be highly interesting to assess whether patients with PTSD caused by childhood vs. those with PTSD caused by adulthood traumata respond differently to chronic multifaceted stressors such as the COVID-19 pandemic.

Speculatively, the higher comorbidity load (here: 2.79 vs. 1.74 F-Diagnoses) and the comparably smaller living spaces (here: < 60m^2^ vs.>  95m^2^) might contribute to their relative increase in vulnerability for stress caused by the pandemic: As PTSD is highly associated with an increased comorbidity of other mental disorders like substance abuse, anxiety disorders or major depression including suicidal ideation [51], coping with stressors may be impeded due to a higher overall symptom load. A smaller living space under 60m^2^ can significantly increase the level of depressive symptoms [52] – this would also potentially impede stress coping and the development of a high level of resilience. However, the relation between smaller living space and stress coping specifically in patients with PTSD has not yet been investigated. Of note, and in contrast to the previously published total sample [23], this could not be verified here as we found no changes in resilience in the PTSD sample. Clearly, the two resilience items of the Goe-BSI inventory employed in this study do not reflect every facet of psychological resilience. As stated by multiple authors (see [53] for an overview) the lacking perception of “own controllability” during the pandemic may indicate a decreased resilience level. This may have been an additional long-term stressor in patients with PTSD during the pandemic, and should be considered for a next version of the Goe-BSI.

The results of this study are limited by various factors, mainly by the small PTSD sample size that classifies it as an exploratory study. These small numbers did not allow Bonferroni-correction between GLM main effects, but, at least, Bonferroni-correction of post-hoc tests. The inclusion of a healthy control group would have been desirable to analyze the impact of the pandemic on mental health in comparison with psychiatric patients, and to improve the overall sample size. Furthermore, our study relied on a monocentric convenience sample which may be considered less representative for a global population of patients with PTSD. The reliance on self-report scales (psychosocial burden, ADNM-20, resilience, psychiatric symptoms) may be subject to biases and should be supplemented by clinician/expert ratings in future studies.

PTSD has been assessed in daily clinical routine by treating clinical experts, which is clearly a strength of our study regarding the fact that many (online) surveys exclusively rely on self-reported diagnoses only. However, systematically using validated diagnostic inventories would add more preciseness to the differentiation of the PTSD and the non-PTSD sample in future studies. So would the additional use of stress coping style questionnaires. In addition, using an approach combining current and retrospective data, we cannot fully exclude the creation of pseudo-trajectories of psychosocial burden and thus the retrospective estimations included in our study must be interpreted with caution [23]. Finally, as we stated previously [23,41], several ICD-10 diagnoses are underrepresented in this convenience sample, in particular diagnoses belonging to the ICD-10 chapters F1 and F5. In sum, the assessment of patients under “pandemic conditions” did largely dictate our inclusion criteria, longitudinal design, and the methods of statistical analysis (see points above). As described, the instruments presented here constituted a compromise to measure a valid stress response pattern and had to be partially developed (Goe-BSI) during the first phase of the pandemic in 2020 – please see our previous studies [23,41] for detailed information (scale development and quality criteria).

Taken together, we provide preliminary and first evidence that, among psychiatric populations, patients with PTSD might be particularly burdened by comorbid AD symptoms and psychosocial stress load in response to the COVID-19 pandemic. The well-known enhanced stress vulnerability of patients with PTSD (e.g., [37,38,50]) might significantly contribute to these findings. Of note, in contrast to the majority of studies on the biological and psychological stress coping in PTSD patients that employed artificially generated stressors such as the Trier Social Stress Test, our study shows that impaired stress coping in patients with PTSD is relevant also in a real-world context. Even though the COVID-19 pandemic has been overcome, this study calls for awareness of the here-proposed exceptionally high vulnerability of patients with PTSD. Other real-world stressors like globally increasing social tensions may have a comparable effect on such patients, but are neither sufficiently investigated nor comparable to a pandemic situation with far-reaching restrictions. The risk of future global pandemics will potentially increase further due to global warming and livestock farming [54], leading to the necessity for policy makers to ensure care of highly vulnerable patients in such times (e.g., via telemedicine).

Based on the experience gathered here, future pandemic-related studies should aim to implement multiple improvements: (1) acquisition of a larger sample size, possibly within a multicenter approach, to also enable the analysis of moderating factors (e.g., trauma type 1 vs. 2); (2) initiation of the first assessment as early as possible, to reduce the number of potentially biased retrospective assessments; (3) implementation of additional measures (e.g., stress coping) and comprehensive measurement of resilience.

## Supporting information

S1 File Dataset of the study. The minimal dataset of the study is provided as SAV-file (SPSS).
(SAV)

## References

[1] WHO Coronavirus (COVID-19) Dashboard. [cited 8 Feb 2023]. Available from: https://covid19.who.int

[2] Bäuerle A, Teufel M, Musche V, Weismüller B, Kohler H, Hetkamp M, et al. Increased generalized anxiety, depression and distress during the COVID-19 pandemic: a cross-sectional study in Germany. J Public Health (Oxf). 2020;42(4):672–8. doi: 10.1093/pubmed/fdaa106 32657323 PMC7454766

[3] Cooke JE, Eirich R, Racine N, Madigan S. Prevalence of posttraumatic and general psychological stress during COVID-19: a rapid review and meta-analysis. Psychiatry Res. 2020;292:113347. doi: 10.1016/j.psychres.2020.113347 32763477 PMC7392847

[4] Gilan D, Röthke N, Blessin M, Kunzler A, Stoffers-Winterling J, Müssig M, et al. Psychomorbidity, resilience, and exacerbating and protective factors during the SARS-CoV-2 pandemic. Dtsch Arztebl Int. 2020;117(38):625–30. doi: 10.3238/arztebl.2020.0625 33200744 PMC7817784

[5] Huang Y, Zhao N. Generalized anxiety disorder, depressive symptoms and sleep quality during COVID-19 outbreak in China: a web-based cross-sectional survey. Psychiatry Res. 2020;288:112954. doi: 10.1016/j.psychres.2020.112954 32325383 PMC7152913

[6] Li J, Yang Z, Qiu H, Wang Y, Jian L, Ji J, et al. Anxiety and depression among general population in China at the peak of the COVID-19 epidemic. World Psychiatry. 2020;19(2):249–50. doi: 10.1002/wps.20758 32394560 PMC7214959

[7] McGinty EE, Presskreischer R, Han H, Barry CL. Psychological distress and loneliness reported by US Adults in 2018 and April 2020. JAMA. 2020;324(1):93–4. doi: 10.1001/jama.2020.9740 32492088 PMC7270868

[8] Petzold MB, Bendau A, Plag J, Pyrkosch L, Mascarell Maricic L, Betzler F, et al. Risk, resilience, psychological distress, and anxiety at the beginning of the COVID-19 pandemic in Germany. Brain Behav. 2020;10(9):e01745. doi: 10.1002/brb3.1745 32633464 PMC7361063

[9] Qiu J, Shen B, Zhao M, Wang Z, Xie B, Xu Y. A nationwide survey of psychological distress among Chinese people in the COVID-19 epidemic: implications and policy recommendations. Gen Psychiatr. 2020;33(2):e100213. doi: 10.1136/gpsych-2020-100213 32215365 PMC7061893

[10] Rossi R, Socci V, Talevi D, Mensi S, Niolu C, Pacitti F, et al. COVID-19 pandemic and lockdown measures impact on mental health among the general population in Italy. Front Psychiatry. 2020;11:790. doi: 10.3389/fpsyt.2020.00790 32848952 PMC7426501

[11] Wang C, Pan R, Wan X, Tan Y, Xu L, McIntyre RS, et al. A longitudinal study on the mental health of general population during the COVID-19 epidemic in China. Brain Behav Immun. 2020;87:40–8. doi: 10.1016/j.bbi.2020.04.028 32298802 PMC7153528

[12] Kramer V, Papazova I, Thoma A, Kunz M, Falkai P, Schneider-Axmann T, et al. Subjective burden and perspectives of German healthcare workers during the COVID-19 pandemic. Eur Arch Psychiatry Clin Neurosci. 2021;271(2):271–81. doi: 10.1007/s00406-020-01183-2 32815019 PMC7437642

[13] Liang Y, Wu K, Zhou Y, Huang X, Zhou Y, Liu Z. Mental health in frontline medical workers during the 2019 novel coronavirus disease epidemic in China: a comparison with the general population. Int J Environ Res Public Health. 2020;17(18):6550. doi: 10.3390/ijerph17186550 32916836 PMC7558595

[14] Liu S, Yang L, Zhang C, Xiang Y-T, Liu Z, Hu S, et al. Online mental health services in China during the COVID-19 outbreak. Lancet Psychiatry. 2020;7(4):e17–8. doi: 10.1016/S2215-0366(20)30077-8 32085841 PMC7129099

[15] Tan BYQ, Chew NWS, Lee GKH, Jing M, Goh Y, Yeo LLL, et al. Psychological impact of the COVID-19 pandemic on health care workers in Singapore. Ann Intern Med. 2020;173(4):317–20. doi: 10.7326/M20-1083 32251513 PMC7143149

[16] Zhang H, Shi Y, Jing P, Zhan P, Fang Y, Wang F. Posttraumatic stress disorder symptoms in healthcare workers after the peak of the COVID-19 outbreak: a survey of a large tertiary care hospital in Wuhan. Psychiatry Res. 2020;294:113541. doi: 10.1016/j.psychres.2020.113541 33128999 PMC7585629

[17] Taquet M, Luciano S, Geddes JR, Harrison PJ. Bidirectional associations between COVID-19 and psychiatric disorder: retrospective cohort studies of 62 354 COVID-19 cases in the USA. Lancet Psychiatry. 2021;8(2):130–40. doi: 10.1016/S2215-0366(20)30462-4 33181098 PMC7820108

[18] Yamamoto V, Bolanos JF, Fiallos J, Strand SE, Morris K, Shahrokhinia S, et al. COVID-19: review of a 21st century pandemic from etiology to neuro-psychiatric implications. J Alzheimers Dis. 2020;77(2):459–504. doi: 10.3233/JAD-200831 32925078 PMC7592693

[19] Castellini G, Cassioli E, Rossi E, Innocenti M, Gironi V, Sanfilippo G, et al. The impact of COVID-19 epidemic on eating disorders: a longitudinal observation of pre versus post psychopathological features in a sample of patients with eating disorders and a group of healthy controls. Int J Eat Disord. 2020;53(11):1855–62. doi: 10.1002/eat.23368 32856333 PMC7461528

[20] Yocum AK, Zhai Y, McInnis MG, Han P. Covid-19 pandemic and lockdown impacts: a description in a longitudinal study of bipolar disorder. J Affect Disord. 2021;282:1226–33. doi: 10.1016/j.jad.2021.01.028 33601700 PMC9754803

[21] Benatti B, Albert U, Maina G, Fiorillo A, Celebre L, Girone N, et al. What happened to patients with obsessive compulsive disorder during the COVID-19 pandemic? a multicentre report from tertiary clinics in northern Italy. Front Psychiatry. 2020;11:720. doi: 10.3389/fpsyt.2020.00720 32793008 PMC7385249

[22] Fiorillo A, Sampogna G, Giallonardo V, Del Vecchio V, Luciano M, Albert U, et al. Effects of the lockdown on the mental health of the general population during the COVID-19 pandemic in Italy: results from the COMET collaborative network. Eur Psychiatry. 2020;63(1):e87. doi: 10.1192/j.eurpsy.2020.89 32981568 PMC7556907

[23] Bartels C, Hessmann P, Schmidt U, Vogelgsang J, Ruhleder M, Kratzenberg A, et al. Medium-term and peri-lockdown course of psychosocial burden during the ongoing COVID-19 pandemic: a longitudinal study on patients with pre-existing mental disorders. Eur Arch Psychiatry Clin Neurosci. 2022;272(5):757–71. doi: 10.1007/s00406-021-01351-y 34825249 PMC8614217

[24] McLean CP, Levy HC, Miller ML, Tolin DF. Exposure therapy for PTSD: a meta-analysis. Clin Psychol Rev. 2022;91:102115. doi: 10.1016/j.cpr.2021.102115 34954460

[25] Fiorillo A, Gorwood P. The consequences of the COVID-19 pandemic on mental health and implications for clinical practice. Eur Psychiatry. 2020;63(1):e32. doi: 10.1192/j.eurpsy.2020.35 32234102 PMC7156565

[26] Kalaitzaki EA, Tsouvelas G, Tamiolaki A, Konstantakopoulos G. Post-traumatic stress symptoms during the first and second COVID-19 lockdown in Greece: Rates, risk, and protective factors. Int J Ment Health Nurs. 2022;31(1):153–66. doi: 10.1111/inm.12945 34658113 PMC8652774

[27] Southwick SM, Charney DS. The science of resilience: implications for the prevention and treatment of depression. Science. 2012;338(6103):79–82. doi: 10.1126/science.1222942 23042887

[28] Maercker A, Heim E, Hecker T, Thoma MV. Social support after traumatism. Nervenarzt. 2017;88(1):18–25. doi: 10.1007/s00115-016-0242-6 27853853

[29] Olff M, Langeland W, Witteveen A, Denys D. A psychobiological rationale for oxytocin in the treatment of posttraumatic stress disorder. CNS Spectr. 2010;15(8):522–30. doi: 10.1017/s109285290000047x 20703199

[30] Vlachos II, Papageorgiou C, Margariti M. Neurobiological trajectories involving social isolation in PTSD: a systematic review. Brain Sci. 2020;10(3):173. doi: 10.3390/brainsci10030173 32197333 PMC7139956

[31] Tortella-Feliu M, Fullana MA, Pérez-Vigil A, Torres X, Chamorro J, Littarelli SA, et al. Risk factors for posttraumatic stress disorder: an umbrella review of systematic reviews and meta-analyses. Neurosci Biobehav Rev. 2019;107:154–65. doi: 10.1016/j.neubiorev.2019.09.013 31520677

[32] Asmundson GJG, Paluszek MM, Landry CA, Rachor GS, McKay D, Taylor S. Do pre-existing anxiety-related and mood disorders differentially impact COVID-19 stress responses and coping? J Anxiety Disord. 2020;74:102271. doi: 10.1016/j.janxdis.2020.102271 32673930 PMC7342169

[33] Murphy D, Williamson C, Baumann J, Busuttil W, Fear NT. Exploring the impact of COVID-19 and restrictions to daily living as a result of social distancing within veterans with pre-existing mental health difficulties. BMJ Mil Health. 2022;168(1):29–33. doi: 10.1136/bmjmilitary-2020-001622 33243764

[34] Forte G, Favieri F, Tambelli R, Casagrande M. COVID-19 Pandemic in the Italian population: validation of a post-traumatic stress disorder questionnaire and prevalence of PTSD symptomatology. Int J Environ Res Public Health. 2020;17(11):4151. doi: 10.3390/ijerph17114151 32532077 PMC7312976

[35] Rajkumari B, Akham N, Konjengbam OK, Pangambam AD, Ningthoujam SD. Post-traumatic stress disorder among COVID-19 survivors in Manipur: a cross-sectional study. J Family Med Prim Care. 2022;11(5):2139–45. doi: 10.4103/jfmpc.jfmpc_1474_21 35800499 PMC9254810

[36] American Psychiatric Association. Diagnostic and statistical manual of mental disorders: DSM-5. 5th ed. Washington, D.C: American Psychiatric Association; 2013.

[37] Wichmann S, Kirschbaum C, Böhme C, Petrowski K. Cortisol stress response in post-traumatic stress disorder, panic disorder, and major depressive disorder patients. Psychoneuroendocrinology. 2017;83:135–41. doi: 10.1016/j.psyneuen.2017.06.005 28623762

[38] Zaba M, Kirmeier T, Ionescu IA, Wollweber B, Buell DR, Gall-Kleebach DJ, et al. Identification and characterization of HPA-axis reactivity endophenotypes in a cohort of female PTSD patients. Psychoneuroendocrinology. 2015;55:102–15. doi: 10.1016/j.psyneuen.2015.02.005 25745955

[39] Schubert CF, Schreckenbach M, Kirmeier T, Gall-Kleebach DJ, Wollweber B, Buell DR, et al. PTSD psychotherapy improves blood pressure but leaves HPA axis feedback sensitivity stable and unaffected: first evidence from a pre-post treatment study. Psychoneuroendocrinology. 2019;100:254–63. doi: 10.1016/j.psyneuen.2018.10.013 30391833

[40] Mazzulo NN. Relations between veterans’ coping strategies and symptoms of PTSD and depression. 2018.

[41] Belz M, Hessmann P, Vogelgsang J, Schmidt U, Ruhleder M, Signerski-Krieger J, et al. Evolution of psychosocial burden and psychiatric symptoms in patients with psychiatric disorders during the Covid-19 pandemic. Eur Arch Psychiatry Clin Neurosci. 2022;272(1):29–40. doi: 10.1007/s00406-021-01268-6 33942148 PMC8092366

[42] Tagay S, Senf W. ETI - Essener Trauma-Inventar. Eine Verfahrensfamilie zur Identifikation von traumatischen Ereignissen und Traumfolgestörungen - Manual. Göttingen: Hogrefe; 2014.

[43] Maercker A, Schützwohl M. Erfassung von psychischen Belastungsfolgen: Die Impact of Event Skala-revidierte Version (IES-R)./ Assessment of post-traumatic stress reactions: The Impact of Event Scale-Revised (IES-R). Diagnostica. 1998;44:130–41. doi: 10.1037/t55092-000

[44] Einsle F, Köllner V, Dannemann S, Maercker A. Development and validation of a self-report for the assessment of adjustment disorders. Psychol Health Med. 2010;15(5):584–95. doi: 10.1080/13548506.2010.487107 20835968

[45] Lorenz L, Bachem RC, Maercker A. The adjustment disorder – new module 20 as a screening instrument: cluster analysis and cut-off values. Int J Occup Environ Med. 2016;7(4):215–20. doi: 10.15171/ijoem.2016.775 27651082 PMC6817961

[46] Cohen J. Statistical power analysis for the behavioral sciences. 2nd ed. Hillsdale, N.J: L. Erlbaum Associates; 1988.

[47] Karatzias T, Knefel M, Maercker A, Cloitre M, Reed G, Bryant RA, et al. The network structure of ICD-11 disorders specifically associated with stress: adjustment disorder, prolonged grief disorder, posttraumatic stress disorder, and complex posttraumatic stress disorder. Psychopathology. 2022;55(3–4):226–34. doi: 10.1159/000523825 35344963

[48] Hughes-Taylor KL. Correctional officers’ response to stress: an exploration of the associations between violence and trauma exposure, coping mechanisms, and PTSD. J Crim Justice. 2021;45(3):123–35. doi: 10.1016/j.jcrimjus.2021.101123

[49] She R, Li L, Yang Q, Lin J, Ye X, Wu S, et al. Associations between COVID-19 work-related stressors and posttraumatic stress symptoms among chinese doctors and nurses: application of stress-coping theory. Int J Environ Res Public Health. 2022;19(10):6201. doi: 10.3390/ijerph19106201 35627736 PMC9140888

[50] Mayer SE, Peckins M, Kuhlman KR, Rajaram N, Lopez-Duran NL, Young EA, et al. The roles of comorbidity and trauma exposure and its timing in shaping HPA axis patterns in depression. Psychoneuroendocrinology. 2020;120:104776. doi: 10.1016/j.psyneuen.2020.104776 32593866 PMC7502500

[51] Galatzer-Levy IR, Nickerson A, Litz BT, Marmar CR. Patterns of lifetime PTSD comorbidity: a latent class analysis. Depress Anxiety. 2013;30(5):489–96. doi: 10.1002/da.22048 23281049

[52] Amerio A, Brambilla A, Morganti A, Aguglia A, Bianchi D, Santi F, et al. COVID-19 lockdown: housing built environment’s effects on mental health. Int J Environ Res Public Health. 2020;17(16):5973. doi: 10.3390/ijerph17165973 32824594 PMC7459481

[53] Vinkers CH, van Amelsvoort T, Bisson JI, Branchi I, Cryan JF, Domschke K, et al. Stress resilience during the coronavirus pandemic. Eur Neuropsychopharmacol. 2020;35:12–6. doi: 10.1016/j.euroneuro.2020.05.003 32446705 PMC7211573

[54] Casadevall A. Pandemics past, present, and future: progress and persistent risks. J Clin Invest. 2024;134(7):e179519. doi: 10.1172/JCI179519 38557492 PMC10977977

